# Effects of exercise mode, energy, and macronutrient interventions on inflammation during military training

**DOI:** 10.14814/phy2.12820

**Published:** 2016-06-07

**Authors:** Stefan M. Pasiakos, Lee M. Margolis, Nancy E. Murphy, Holy L. McClung, Svein Martini, Yngvar Gundersen, John W. Castellani, James P. Karl, Hilde K. Teien, Elisabeth H. Madslien, Pal H. Stenberg, Andrew J. Young, Scott J. Montain, James P. McClung

**Affiliations:** ^1^ Military Nutrition DivisionUS Army Research Institute of Environmental MedicineNatickMassachusetts; ^2^ Norwegian Defence Research EstablishmentKjellerNorway; ^3^ Thermal Mountain and Medicine DivisionUS Army Research Institute of Environmental MedicineNatickMassachusetts; ^4^ General Defence Material/Catering and Combat Feeding SectionNorwegian NavyRødskiferveienNorway

**Keywords:** Endurance exercise, energy deficit, iron status, macronutrients

## Abstract

Load carriage (LC) exercise may exacerbate inflammation during training. Nutritional supplementation may mitigate this response by sparing endogenous carbohydrate stores, enhancing glycogen repletion, and attenuating negative energy balance. Two studies were conducted to assess inflammatory responses to acute LC and training, with or without nutritional supplementation. Study 1: 40 adults fed eucaloric diets performed 90‐min of either LC (treadmill, mean ± SD 24 ± 3 kg LC) or cycle ergometry (CE) matched for intensity (2.2 ± 0.1 VO_2peak_ L min^−1^) during which combined 10 g protein/46 g carbohydrate (223 kcal) or non‐nutritive (22 kcal) control drinks were consumed. Study 2: 73 Soldiers received either combat rations alone or supplemented with 1000 kcal day^−1^ from 20 g protein‐ or 48 g carbohydrate‐based bars during a 4‐day, 51 km ski march (~45 kg LC, energy expenditure 6155 ± 515 kcal day^−1^ and intake 2866 ± 616 kcal day^−1^). IL‐6, hepcidin, and ferritin were measured at baseline, 3‐h post exercise (PE), 24‐h PE, 48‐h PE, and 72‐h PE in study 1, and before (PRE) and after (POST) the 4‐d ski march in study 2. Study 1: IL‐6 was higher 3‐h and 24‐h post exercise (PE) for CE only (mode × time, *P *<* *0.05), hepcidin increased 3‐h PE and recovered by 48‐h, and ferritin peaked 24‐h and remained elevated 72‐h PE (*P *<* *0.05), regardless of mode and diet. Study 2: IL‐6, hepcidin and ferritin were higher (*P *<* *0.05) after training, regardless of group assignment. Energy expenditure (*r *=* *0.40), intake (*r *=* *−0.26), and balance (*r *=* *−0.43) were associated (*P* < 0.05) with hepcidin after training. Inflammation after acute LC and CE was similar and not affected by supplemental nutrition during energy balance. The magnitude of hepcidin response was inversely related to energy balance suggesting that eating enough to balance energy expenditure might attenuate the inflammatory response to military training.

## Introduction

Endurance exercise elicits an increase in IL‐6, a proinflammatory cytokine that, in turn, triggers hepatic release of hepcidin, a regulator of iron status (Peeling 2010; Peeling et al. 2014). A single bout of traditional weight‐bearing and nonweight‐bearing endurance exercise acutely increases circulating IL‐6 and hepcidin concentrations to a similar degree (Sim et al. 2013, 2014a). However, repeated bouts of weight‐bearing endurance exercise (i.e., training) produce a chronic inflammatory response that elevates basal hepcidin levels to a greater extent than repeated bouts of nonweight‐bearing endurance exercise (Sim et al. 2014b). The authors of that study suggested that the elevated levels of hepcidin may alter iron status in athletes participating in weight‐bearing exercise training programs (Sim et al. 2014b). The inflammatory responses to military training may exceed those observed after traditional endurance exercise training, as the unaccustomed activities encountered during military training differ greatly from traditional exercise. For example, load carriage (LC) is not typically performed during traditional exercise training, but is commonly performed for prolonged periods at low to moderate intensities with loads ranging from 20 to 60 kg during military training (Nindl et al. 2013). Whether the physiological strain of LC elicits an inflammatory and hepcidin response that exceeds responses produced during training employing traditional endurance exercise has not been determined.

Military training can produce severe energy deficits that deplete endogenous energy stores (Margolis et al. 2014). Depleted endogenous energy stores may exacerbate inflammation and the hepcidin response to exercise, as low glycogen status upregulates postexercise skeletal muscle IL‐6 gene expression (Keller et al. 2001; Steensberg et al. 2001) and increases plasma levels of IL‐6 (Steensberg et al. 2000). It has been hypothesized that carbohydrate supplementation might attenuate postexercise inflammation by limiting glycogen depletion during sustained endurance exercise and enhancing glycogen repletion during recovery, although the data supporting this hypothesis are largely equivocal (Cox et al. 2008, 2010; Sim et al. 2012; Badenhorst et al. 2015). Some studies suggest that combining whey protein with carbohydrate attenuates the inflammatory response to exercise (Kerasioti et al. 2013), although other studies fail to substantiate such an effect (Nelson et al. 2013; Rowlands et al. 2008). Of note, each of these studies was performed in laboratory settings under controlled periods of energy balance. To what extent carbohydrate, protein, or combined supplementation attenuates the inflammatory responses to arduous military training (McClung et al. 2013; Margolis et al. 2014) has not been tested.

In this study, two experiments were conducted to assess inflammation and hepcidin responses to military unique endurance exercise tasks, with or without supplemental nutrition. Study 1 compared inflammatory and hepcidin responses to LC and intensity‐matched, nonweight‐bearing exercise, cycle ergometry (CE), and evaluated whether consuming a combined protein and carbohydrate supplement mitigated the inflammatory response. We hypothesized that the postexercise inflammatory and hepcidin responses to acute LC would be more pronounced than traditional CE, and that combined carbohydrate and protein supplementation would attenuate the acute response. Study 2 assessed the efficacy of a carbohydrate‐ or protein‐based nutrition intervention on inflammation and hepcidin responses during a multi‐day military training operation in which participants performed repeated days of LC and performed activities that produced severe energy deficits. We expected carbohydrate‐based supplemental nutrition to mitigate inflammatory and hepcidin responses during multiday military training to a greater extent than protein‐based supplementation.

## Methods

### Study 1: Volunteers and experimental design

Forty adults (37 males and 3 females) participated in this randomized, double‐blind, placebo‐controlled study after providing informed, written consent from October 2012 to November 2013 (Pasiakos et al. 2015). All study procedures were conducted at the US Army Research Institute of Environmental Medicine (USARIEM, Natick, MA). Volunteers were military personnel from the US Army Natick Research, Development and Engineering Center, Human Research Volunteer recruit platoon, and civilians from the local area. Volunteers were required to be between the ages of 18–39 years, weight stable (±2 kg for a period of 2 months), physically fit (peak oxygen uptake, VO_2peak_ 40–60 mL kg^−1^ min^−1^), and have a body mass index (BMI) between 22 and 29 kg m^−2^. A medical screening was also conducted to ensure that potential volunteers could safely participate in the study. This study was approved by the Institutional Review Board at USARIEM and registered at www.clinicaltrials.gov as NCT01714479.

**Figure 1:**
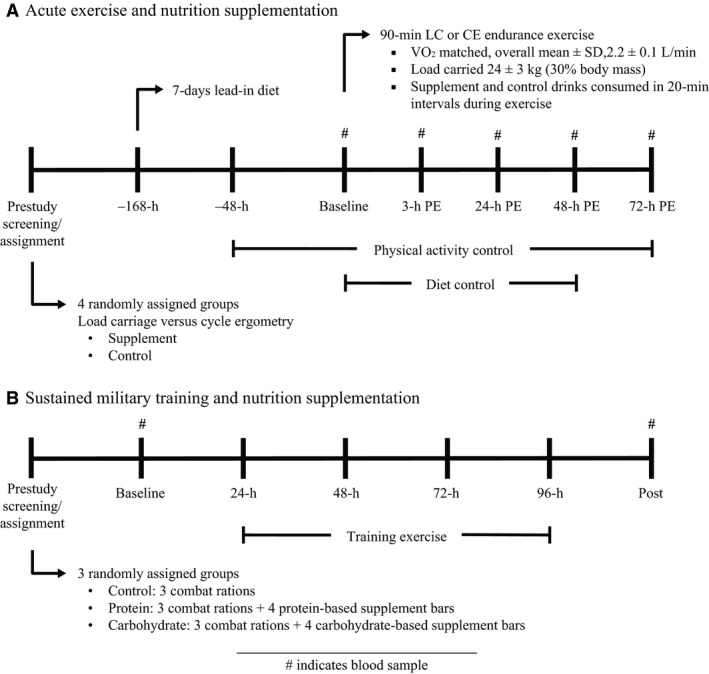
Study designs. (A) Experimental design for assessing the inflammatory responses to acute, 90‐min load carriage (LC) or cycle ergometry (CE) intensity‐matched exercise bouts, with or without combined essential amino acid and carbohydrate supplementation. Biochemical assays were performed using blood collected at baseline, 3‐h postexercise (PE), 24‐h PE, 48‐h PE, and 72‐h PE. Dietary intake was individually prescribed to maintain body mass and provided to research volunteers as US military combat rations. (B) Experimental design for assessing the effects of carbohydrate and protein supplementation on inflammation before (PRE) and immediately after (POST) completing a 4‐day arctic military training operation.

Volunteers were randomly assigned to one of four experimental groups (Fig. 1A). All four groups performed a single 90‐min exercise bout. Two groups performed nonweight‐bearing, traditional endurance exercise (CE) and the other two performed LC (i.e., a military‐like, occupational exercise) exercise. One of each of the exercise groups received combined essential amino acid and carbohydrate supplement (SUPP, 223 kcal) drinks to consume during exercise, and the other groups received flavor‐matched, nonnutritive control (CON, 22 kcal, 5 g carbohydrate) drinks. The essential amino acid/carbohydrate treatment provided 10 g of essential amino acids (0.7 g histidine, 0.7 g isoleucine, 3.6 g leucine, 1.2 g lysine, 0.3 g methionine, 1.4 g phenylalanine, 1.0 g threonine, and 1 g valine) and 46 g of carbohydrate (maltodextrin and fructose at a 2:1 ratio). The composition of the SUPP treatment was based on previous work demonstrating a muscle protein synthetic advantage of consuming small doses of leucine‐enriched essential amino acids during steady‐state exercise (Pasiakos et al. 2011). However, the treatment also included carbohydrate to test a palatable, eat‐on‐the move, combat ration recovery item that provides not only essential amino acids, but also energy in the form of carbohydrate to sustain activity during military operations (Jeukendrup 2014). Inflammation (IL‐6), hepcidin, and ferritin, because ferritin is also induced by inflammation, were assessed at baseline (preexercise), postexercise, and once daily during a 72‐h recovery period. Creatine kinase (CK) was measured as a surrogate marker of muscle damage to differentiate skeletal muscle strain between LC and CE.

### Diet and physical activity

Dietary intake and physical activity were controlled to minimize any potential confounding effects on outcome variables. Dietary intake was individually prescribed based on 3‐day diet and activity records at baseline and dietary compliance was confirmed by conducting 24‐h dietary recalls every 2 days during a 7‐day prestudy lead‐in phase (Food Processor SQL^®^, version 10, ESHA Research, Salem, OR). Overall (mean ± SD) energy (2607 ± 468 kcal day^−1^), carbohydrate (344 ± 78 g day^−1^), protein (111 ± 23 g day^−1^), and fat (92 ± 26 g day^−1^) intakes were similar across groups, and body mass was stable during the lead‐in phase (Pasiakos et al. 2015). Eucaloric diets were then standardized across groups beginning with dinner the evening before the experimental exercise session, and ending with dinner 48‐h post exercise. Volunteers were instructed to maintain activity levels reported at baseline for the first 5 days of the lead‐in phase. All resistive and endurance‐type physical activity was then prohibited from 48‐h before the experimental exercise session until after the 72‐h data collection period was completed.

### Experimental load carriage and cycle ergometry

Volunteers performed LC by walking on a treadmill while wearing a weighted vest equivalent to 30% of baseline body mass. A Lode (BV, Netherlands) ergometer was used for the CE exercise bouts. Baseline VO_2peak_ and associated heart rates at maximal and submaximal levels were used to establish a target exercise intensity of 2.4 L min^−1^ for both the LC and CE trials. Absolute exercise intensity was matched between LC and CE by adjusting the speed and grade for LC and power for CE. By matching the intensity and, ultimately, energy cost, the effects of possible differences in mechanical force and contractile properties of LC and CE from the relative intensity and energy cost of the exercise bout were isolated. A 90‐min familiarization trial was conducted at least 1 week before the experimental session to ensure the accuracy of the exercise prescription and the ability of the volunteer to complete the prescribed exercise bout. Heart rate was monitored continuously, and indirect calorimetry (ParvoMedics, Sandy, UT) was used to verify exercise intensity (15‐min intervals). Workloads were adjusted to maintain the desired exercise intensity.

The experimental LC and CE sessions were conducted in the morning following a 12‐h fast. Volunteers began the 90 min intensity‐matched LC or CE exercise bout after baseline blood sampling. Exercise intensity was verified (and adjusted accordingly) every 30‐min and adjustments were made based on indirect calorimetry. Overall intensity was not different between groups: oxygen uptake was 2.2 ± 0.1 L min^−1^, energy expenditure was 1000 ± 60 kcal∙90‐min^−1^ and average load carried for LC groups was 24 ± 3 kg. Volunteers consumed equal volumes (500 mL total, 125 mL per serving) of either the SUPP or flavor‐matched, nonnutritive CON drinks in 30‐min intervals, beginning at the start of the exercise session and ending after completing the 90‐min bout. Study staff and volunteers were blinded and supplements were prepared and coded by an independent third party (Combat Feeding Directorate, US Army Natick Soldier Systems Center, Natick, MA) to eliminate bias. Postexercise (PE) and recovery blood samples were obtained 3‐h, 24‐h, 48‐h, and 72‐h to assess temporal changes in study outcomes.

### Study 2: volunteers and experimental design

Seventy‐three Norwegian Soldiers (71 males and 2 females) stationed in Skjold, NO participated in this randomized controlled study after providing informed, written consent (Margolis et al. 2016). Volunteers were scheduled to participate in a 4‐day winter military training program as part of their routine training in January of 2015. This study was approved by the Human Use Review Committee at the US Army Research Institute of Environmental Medicine (Natick, MA, USA) and the Regional Committees for Medical and Health Research Ethics (REK sør‐øst, Oslo, NO). This trial was registered at www.clinicaltrials.gov as NCT02327208.

Volunteers were block randomized by body mass to one of three dietary treatment groups for the duration of the training program: three Norwegian arctic combat rations alone (control, CON), three rations plus four whey protein‐based snack products (PRO), and three rations plus four carbohydrate‐based snack products (CHO). The total number of rations provided was based on local Norwegian Army command policy for this specific training program. Volunteers began consuming the daily allotment of three Norwegian arctic combat rations (provided by study staff) 2 days before the start of the training program. Three combat rations provided 3487 kcal day^−1^, 141 g day^−1^ protein, 435 g day^−1^ carbohydrate, and 126 g day^−1^ fat if consumed in their entirety. Volunteers assigned to PRO and CHO were provided four snack bars beginning on day one of the training program and were instructed to consume the bars either between meals, after a prolonged period of cross‐country skiing, or before bed. Four PRO snack products provided approximately 1062 kcal day^−1^, 85 g day^−1^ whey protein, 102 g day^−1^ carbohydrate, and 35 g day^−1^ fat, whereas four CHO products provided approximately 1058 kcal day^−1^, 11 g day^−1^ whey protein, 189 g day^−1^ carbohydrate, and 29 g day^−1^ fat, if consumed in their entirety (Margolis et al. 2016). The snack bars were manufactured by the Combat Feeding Directorate (Natick, MA) and designed to be isocaloric and similar in serving size, taste, and textural qualities to blind volunteers and study staff (only on treatment groups) to eliminate bias. No additional food or dietary supplements were permitted for any group and volunteers were instructed to only eat the bars and rations they received and not trade or share the bars with other Soldiers. Fasted blood samples (0500–0700 h) obtained before (PRE) and within 8 h of completing the 4‐day training program (POST) were used to assess CK, IL‐6, hepcidin, and ferritin (Fig. 1B).

### 4‐day winter training program and experimental dietary intake

The training program consisted of sustained periods of low‐to‐moderate intensity physical activity, primarily LC (51 km cross‐country skiing), with intermittent periods of rest and more intense activity. Volunteers carried approximately 45 kg of gear in a weighted pack while skiing during the 4‐day program and were encouraged to maintain hydration by drinking fluids ad libitum.

Energy and macronutrient intake were determined using ration‐specific food logs. Volunteers were provided with cards containing a list of all the items in the provided rations, and were trained to record the percentage of each item and number of snack bars consumed. Food logs were collected daily, with trained Registered Dietitians verifying items consumed with each participant. The amount of each ration item consumed was subtracted from the known initial amount to calculate energy, protein, carbohydrate, and fat intake. These data were used in combination with doubly labeled water (DLW) estimates of energy expenditure to calculate energy balance. In brief, energy expenditure was assessed in a subset of volunteers (*n* = 14 per group) using DLW (0.23 g of H_2_^18^O per total body water (TBW) (kg) and 0.15 g of ^2^H_2_O per TBW (kg); Sigma‐Aldrich, St. Louis, MO). Volunteers were consumed the DLW at baseline, daily urine samples (morning void) were collected to determine isotopic elimination rates during the training exercise, and used to calculate energy expenditure (Schoeller et al. 1986). To account for the influence of fat‐free mass on energy expenditure, regression modeling, with fat‐free mass (measured by skinfold) as a covariate, was conducted. The predictive equation generated [kcal day^−1^ = 1291 + (69.1 × fat‐free mass)] was also used to estimate energy expenditure for the study volunteers not dosed with DLW (Redman et al. 2014; Margolis et al. 2016). Dietary intake, energy expenditure, and resulting energy balance have been reported previously (Margolis et al. 2016), but are included in this manuscript in order to explore associations between diet and markers of inflammation, muscle damage, and iron status.

### Biological analyses

Blood samples, with the exception of 3‐h post exercise (PE) for study 1, were collected after an overnight fast by antecubital venipuncture (Vacutainer; Becton Dickson, Franklin Lakes, NJ). Serum was isolated, frozen, and shipped on dry ice to the Pennington Biomedical Research Center (Baton Rouge, LA) for analysis of CK (Beckman Coulter DXC 600 Pro, Beckman Coulter, Brea CA), IL‐6 (Milliplex MAP; Millipore, Billerica, MA), hepcidin (DRG International, Inc, Springfield, NJ) and ferritin (Siemens Medical Solutions USA Inc, Malvern, PA).

### Statistical analyses

One‐way ANOVA was used to confirm homogeneity of groups within each study. All variables were examined for normality. Variables exhibiting nonnormal distributions were transformed for analysis. Mixed model repeated measures ANOVA was used to determine main and interactive effects of exercise mode (LC vs. CE), drink (EAA vs. CON), and time (baseline, 3‐h, 24‐h, 48‐h, and 72‐h post exercise) for study 1. Akaike's information criterion was used to determine the appropriate covariance model (Burnham and Anderson 2002). Repeated measures ANOVA was performed to assess dietary treatment (CON, CHO, and PRO) and time (PRE and POST) for study 2. Bonferroni adjustments were used for post hoc comparisons if interactions were observed. Associations between IL‐6, hepcidin, ferritin, and the change from PRE and POST were determined using Pearson correlation coefficients for the complete dataset. Pearson correlation coefficients were also determined to assess relationships between dietary intake (carbohydrate, protein, and fat), energy expenditure, and energy balance with IL‐6, hepcidin, ferritin, and the change from PRE and POST. Significance was *P *<* *0.05 and data were analyzed using SPSS (Version 21.0, 2010, SPSS Inc, Chicago, IL) and expressed as means ± SD.

## Results

### Study 1

Baseline volunteer characteristics were not different between dietary treatment groups (Table 1) (Pasiakos et al. 2015). Dietary intake during the recovery phase was similar between dietary treatment groups and averaged 2788 ± 239 kcal day^−1^, 112 ± 14 g day^−1^ protein, 373 ± 28 g day^−1^ carbohydrate, and 97 ± 10 g day^−1^ fat (Table 2).

**Table 1:** Volunteer characteristics for study 1 and 2

	Age (years)	Height (cm)	Body mass (kg)	BMI (kg m^−2^)	Peak VO_2_ mL kg^−1^min^−1^
Study 1					
LC‐CON	24 ± 5	177 ± 8	77 ± 10	25 ± 3	51 ± 5
LC‐SUPP	22 ± 3	178 ± 5	81 ± 10	25 ± 3	51 ± 4
CE‐CON	22 ± 4	175 ± 8	78 ± 11	25 ± 2	50 ± 4
CE‐SUPP	22 ± 2	177 ± 7	84 ± 10	26 ± 2	49 ± 4
Study 2					
CON	19 ± 2	182 ± 7	77 ± 6	23 ± 2	**–**
CHO	20 ± 1	180 ± 6	78 ± 9	24 ± 2	**–**
PRO	20 ± 1	184 ± 7	78 ± 9	23 ± 2	**–**

Data are means ± SD. *N* = 10 per group for LC‐CON (load carriage + nonnutritive control), LC‐SUPP (load carriage + essential amino acid and carbohydrate supplement), CE‐CON (cycle ergometry + nonnutritive control), and CE‐SUPP (cycle ergometry+ essential amino acid and carbohydrate supplement). *N* = 18 for CON (control, 3 rations only), 27 for CHO (3 rations + 4 carbohydrate‐based snacks), and 28 for PRO (3 rations + 4 protein‐based snacks). Peak VO_2_ (aerobic capacity assessed for experiment 1 only using indirect calorimetry, ParvoMedics, Sandy, UT). Data were analyzed for homogeneity using a one‐way ANOVA. No differences were observed between groups.

BMI, body mass index; CE, cycle ergometry.

**Table 2:** Energy and macronutrient intake for the 72‐h recovery period in study 1 and 4‐d arctic military training operation in study 2

	Energy kcal day^−1^	Carbohydrate g day^−1^	Protein g day^−1^	Fat g day^−1^
Study 1				
LC‐CON	2769 ± 298	373 ± 39	108 ± 15	97 ± 12
LC‐SUPP	2804 ± 201	376 ± 20	114 ± 12	96 ± 10
CE‐CON	2714 ± 230	361 ± 28	109 ± 16	97 ± 10
CE‐SUPP	2858 ± 231	382 ± 26	118 ± 14	99 ± 10
Study 2				
CON	2506 ± 410^ac^	312 ± 47^a^	100 ± 15^b^	91 ± 20^a^
CHO	3131 ± 632^b^	434 ± 86^b^	98 ± 22^b^	107 ± 24^a^
PRO	2824 ± 599^ab^	321 ± 77^a^	148 ± 25^a^	102 ± 23^a^

Data are means ± SD. *N* = 10 per group for LC‐CON load carriage + nonnutritive control), LC‐SUPP (load carriage + essential amino acid and carbohydrate supplement), CE‐CON (cycle ergometry + nonnutritive control), and CE‐SUPP (cycle ergometry+ essential amino acid and carbohydrate supplement). *N* = 18 for CON (control, 3 rations only), 27 for CHO (3 rations + 4 carbohydrate‐based snacks), and 28 for PRO (3 rations + 4 protein‐based snacks). A one‐way ANOVA was used to determine differences in dietary intake across groups for each study. Data within a column not sharing the same superscript are different, *P *<* *0.05.

**Figure 2:**
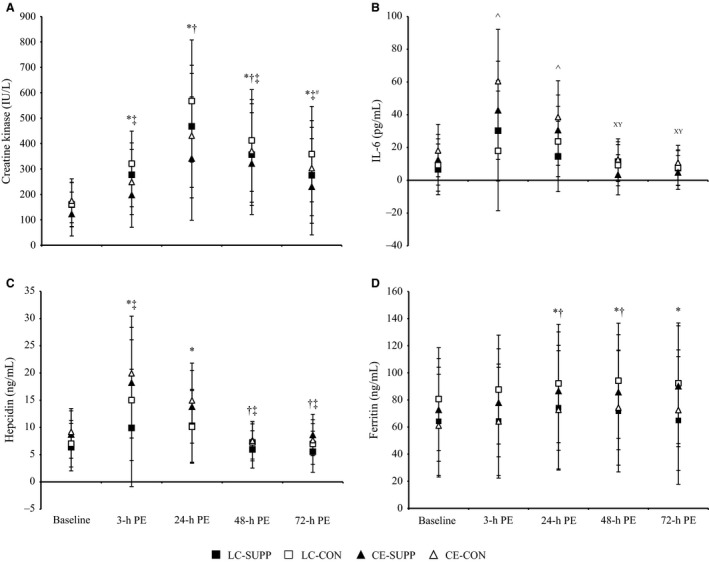
Inflammatory responses to load carriage and cycle ergometry exercise, with or without combined essential amino acid and carbohydrate supplementation. Data are mean ± SD, *n* = 10 per group. Repeated measures ANOVA was used to determine time, exercise mode, and dietary treatment effects and their interactions. Overall mean different from baseline*, 3‐h PE^†^, 24‐h PE^‡^, and 48‐h PE^#,^ main effects of time, *P *<* *0.05. Overall mean for CE 3‐h and 24‐h PE^˄^ different than baseline and overall mean for CE 48‐h and 72‐h different from CE 24‐h PE^xy^, mode‐by‐time, *P *<* *0.05. PE, postexercise, CE, conventional endurance exercise; LC, load carriage; CON, control; and SUPP, essential amino acid + carbohydrate supplement.

Regardless of dietary treatment, CK concentrations increased in response to both LC and CE, and peaked 24‐h PE, without returning to baseline levels during the study period (main effect of time, *P *<* *0.05, Fig. 2A). Dietary treatment did not affect IL‐6 responses to LC or CE; IL‐6 concentrations were elevated 3‐h and 24‐h PE following CE, but not statistically changed following LC (mode × time, *P *<* *0.05, Fig. 2B). Hepcidin was elevated at 3‐h and 24‐h PE following both LC and CE, independent of dietary treatment, and returned to baseline levels by 48‐h (time main effect, *P *<* *0.05, Fig. 2C). Ferritin concentrations were elevated 24‐h PE and remained elevated throughout the 72‐h PE study period, independent of dietary treatment or exercise mode (main effect of time, *P *<* *0.05, Fig. 2D).

### Study 2

Although sample size differed between the PRO (*n* = 28) and CHO (*n* = 27) compared to the CON (*n* = 18), volunteer characteristics across the three dietary treatment groups were not different at the start of the study period (Table 1). As intended and previously reported (Margolis et al. 2016), energy and macronutrient intake during the training exercise differed across experimental and control groups (Table 2). Energy intake in the CHO group was greater (*P *<* *0.05) than CON and similar to PRO. Carbohydrate intake was greater (*P *<* *0.05) in CHO compared to PRO and CON. Protein intake was greater (*P *<* *0.05) in the PRO group compared to CHO and CON. Total energy expenditure was similar across groups and averaged 6155 ± 515 kcal day^−1^, resulting in a mean loss of 2.7 ± 1.2 kg body mass and an energy deficit of 55%; the reductions in body weight and energy balance were not different across groups (Margolis et al. 2016).

**Figure 3:**
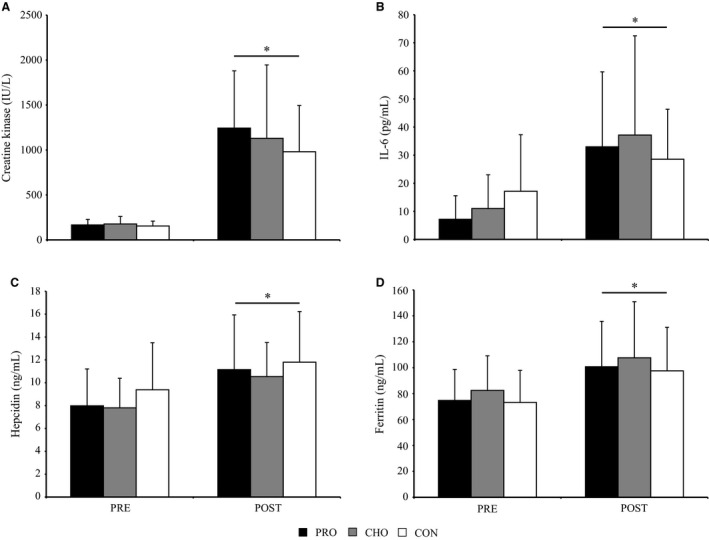
Effects of carbohydrate and protein supplementation on inflammation and associated outcomes before (PRE) and after (POST) a 4‐day arctic military training operation. Data are mean ± SD for CON (control, *n* = 18), CHO (carbohydrate, *n* = 27), and PRO (protein, *n* = 28) dietary groups (G). A repeated measures ANOVA was used to determine time main effects and time by group interactions. Overall mean for postarctic military training (POST) different than baseline (PRE)*, *P *<* *0.0001. CK, creatine kinase, and IL‐6, interleukin‐6.

Circulating CK was nearly six times greater (main effect of time, *P *<* *0.05) POST versus PRE (Fig. 3A). IL‐6 was greater (main effect of time, *P *<* *0.05, Fig. 3B) POST versus PRE and hepcidin was greater (main effect of time, *P *<* *0.05, Fig. 3C) POST versus PRE. Similarly, ferritin increased (time main effect, *P *<* *0.05) with training (Fig. 3D). Dietary treatment did not significantly impact CK, IL‐6, hepcidin, or ferritin responses to training.

**Figure 4:**
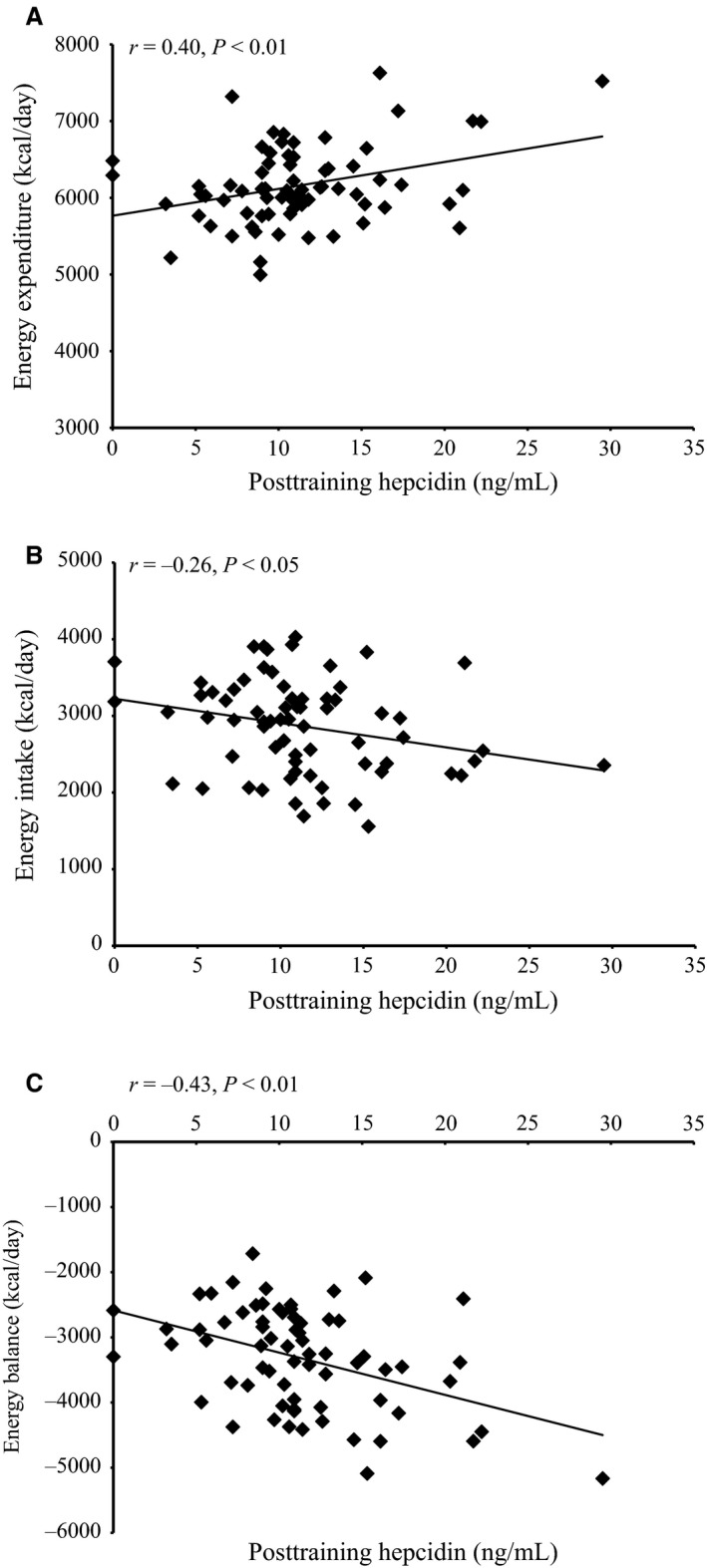
Relationships between energy expenditure (A), energy intake (B), energy balance (C), and hepcidin concentrations after (POST) completing a 4‐day arctic military training operation using Pearson correlation coefficients.

Hepcidin concentrations after the 4‐d training operation were associated (*P *<* *0.05) with total daily energy expenditure (*r *=* *0.40), energy intake (*r *=* *−0.26) and resulting energy balance (*r *=* *−0.43) (Fig. 4A–C). There were no significant associations between IL‐6, hepcidin, and ferritin, or between changes in these variables from PRE to POST. There were no significant associations between carbohydrate and protein intake with IL‐6, hepcidin, and the change in these variables during training.

## Discussion

The inflammatory response to physical activity has been characterized in civilian (Roecker et al. 2005) and military populations (McClung et al. 2013), and may result in poor iron status by increasing hepatic expression of hepcidin (McClung et al. 2009b). The objective of this study was to clarify the impact of exercise mode and to explore the efficacy of energy and macronutrient interventions for attenuating the inflammatory responses to physical activities of relevance to the military. In sum, intensity‐matched LC and traditional, nonweight‐bearing CE caused comparable changes in IL‐6, hepcidin and ferritin over a 72‐h period. Consuming a combined carbohydrate and protein (essential amino acids) supplement during the exercise bout did not attenuate inflammation, and macronutrient manipulation did not affect their responses to multiday military training. However, the increase in hepcidin levels postmilitary training was positively associated with energy expenditure and negatively associated with energy balance. These data reinforce that the hepcidin response to highly demanding military training are, in part, dependent on nutritional status, in particular, energy status (McClung et al. 2009b, 2013; Yanovich et al. 2015).

The unaccustomed physical activities performed during military training, including sustained low‐to‐moderate intensity LC exercise (McClung et al. 2013; Nindl et al. 2013), may contribute to long‐term decrements in iron status (Karl et al. 2010; McClung et al. 2009a, 2009b; Yanovich et al. 2015). Like others (Nieman et al. 1998), we suspected that the eccentric forces generated during LC (i.e., weight‐bearing exercise) would exceed CE, and produce more muscle damage and greater increases in IL‐6 and hepcidin. However, with few exceptions, the effects of LC and CE exercise on CK, IL‐6 and hepcidin were not different. We suspect that the variability in IL‐6 contributed to our inability to detect a statistical increase in IL‐6 after LC, because numerically, the magnitude of increase in IL‐6 after LC was similar to the increase observed after CE. These data indicate that carrying additional weight during low‐to‐moderate intensity endurance exercise was not quantitatively more damaging and did not cause more inflammation than nonweight‐bearing, traditional endurance exercise when the absolute intensity and work performed during the 90‐min sessions were matched. These findings are supported by others (Ostrowski et al. 2000; Sim et al. 2013; Toft et al. 2002), including Sim et al. (2013), who demonstrated that increases in IL‐6 and hepcidin levels 3‐h after completing continuous low (45 min at 65% VO_2peak_) or intermittent, high intensity (8 × 3‐min intervals at 85% VO_2peak_) running and CE were similar across modalities and intensities. Studies comparing longer duration (2.5‐h), higher intensity (75% VO_2max)_ treadmill running and CE exercise bouts have reported similar findings (Nieman et al. 1998). Our data confirm the transient effects of low‐to‐moderate intensity endurance exercise on IL‐6 and hepcidin, and indicate that when the absolute intensities are matched, exercise mode may not augment the inflammatory and hepcidin response to endurance exercise.

Low carbohydrate availability and muscle glycogen depletion upregulate intramuscular gene expression (Keller et al. 2001; Steensberg et al. 2001) and circulating levels of IL‐6 (Steensberg et al. 2000). The relationship between carbohydrate availability and muscle glycogen provided the basis for studies attempting to attenuate postendurance exercise inflammation using carbohydrate supplementation alone (Nieman et al. 1998; Cox et al. 2008; Kerasioti et al. 2013; Sim et al. 2012; Badenhorst et al. 2015) or combined with protein (Kerasioti et al. 2013; Nelson et al. 2013; Rowlands et al. 2008). In this study, a combined carbohydrate (46 g) and essential amino acid (10 g) supplement was provided during the 90‐min LC and CE exercise bouts, and although the intervention was originally designed to maximize muscle recovery (Pasiakos et al. 2015), the levels of carbohydrate and protein provided were similar to other studies specifically designed to assess inflammation (Nelson et al. 2013; Sim et al. 2012). Sim et al. (2012) provided approximately 51 g of carbohydrate in a 6% solution at a dose of 3 mL kg^−1^ every 20‐min during a 90‐min treadmill run (75% VO_2peak)_ and found no differences in IL‐6 and hepcidin 3‐h and 24‐h post exercise compared to a placebo. Nelson et al. (2013) compared a leucine‐enriched protein (7.5 and 20 g), carbohydrate (89 g) and fat (22 g) supplement to an isocaloric carbohydrate (119 g) and fat (22 g) supplement provided once every 3‐h after intense CE for six consecutive days and found no benefit of either supplement on postexercise cytokine expression. The lack of any measureable anti‐inflammatory benefit in this study conflicts with our hypothesis, and although we provided similar or lower amounts of carbohydrate and protein compared to previous studies (Sim et al. 2012; Nelson et al. 2013), we expected inflammation and subsequent hepcidin release to be greater for LC, providing an inflammatory state that may be sensitive to the anti‐inflammatory potential of carbohydrate and protein. However, postexercise and recovery measures of inflammation and hepcidin were similar between the intensity‐matched modes of exercise, an effect likely attributed to the highly controlled period of energy balance in which studies were conducted.

In study 2, the 4‐day military training comprised of daily, sustained LC exercise produced high energy expenditure, severe energy deficit, muscle damage, and increased IL‐6, hepcidin, and ferritin concentrations. The extent to which these parameters changed during the 51‐km ski march was similar to a previous winter training study (McClung et al. 2013; Margolis et al. 2014). We hypothesized that the level of the energy deficit produced during training would diminish endogenous carbohydrate availability and exacerbate the inflammatory response (Cox et al. 2008). Consistent with our hypothesis, energy expenditure and negative energy balance explained a significant portion of the variance in hepcidin levels following the military training activity. As such, we expected that providing additional energy in the form of carbohydrate‐ and protein‐based bars would effectively increase energy intake, spare endogenous carbohydrate availability, and attenuate increases in IL‐6 and hepcidin. However, there were no differences in IL‐6 and hepcidin between groups nor were associations observed between the types of macronutrient consumed and IL‐6, hepcidin, and ferritin levels. However, there was an inverse relationship between the magnitude of energy imbalance and the hepcidin response to the ski march. Although others have assessed cytokine responses to dietary manipulations during military training (Kramer et al. 1997), no studies have directly assessed the potential anti‐inflammatory effects of eating enough to preserve energy balance during short‐term, metabolically demanding military training.

Experimental control and sample size are strengths of the present studies. Dietary intake and exercise intensities for LC and CE trials were highly controlled, and although the cross‐sectional design may be considered a limitation, the control of diet‐ and exercise‐related confounders diminish the negative effects of between group variability. Inability to fully quantify the volume and type of physical exercise performed during the 4‐day ski march may also be a limitation. Finally, the designs of these studies do not allow us to characterize whether added energy intake would reduce the magnitude of inflammation.

In conclusion, these studies are the first to characterize the effects of a military endurance‐type exercise mode (i.e., load carriage) performed once in a controlled setting and repeatedly during a strenuous, energy demanding, real‐world military training exercise. That findings are consistent with previous studies suggests that military endurance‐type exercise is not, in and of itself, more inflammatory than metabolically matched conventional endurance exercise; independent of supplemental nutrition. Furthermore, consuming supplemental carbohydrate and protein during short‐term training did not attenuate the inflammatory response when there was substantial energy deficit. That energy intake, expenditure, and balance were associated with elevations in hepcidin concentrations said the magnitude of hepcidin response was inversely related to energy deficit, suggesting that eating enough to more closely meet energy expenditure, might attenuate potential declines in iron status that result from repeated exposure to unaccustomed activities. Overall, these studies provide novel and practical information for future studies designed to improve military field feeding by manipulating policy and combat ration development to promote energy balance and attenuate the inflammatory response to military training.

## Conflict of Interest

The investigators adhered to the policies for protection of human subjects as prescribed in Army Regulation 70–25, and the research was conducted in adherence with the provisions of 32 CFR part 219. The opinions or assertions contained herein are the private views of the authors and are not to be construed as official or as reflecting the views of the Army or the Department of Defense. Any citations of commercial organizations and trade names in this report do not constitute an official Department of the Army endorsement of approval of the products or services of these organizations.
